# Different battle, same strategy: DNA viruses also block plant autophagy

**DOI:** 10.1093/plphys/kiad266

**Published:** 2023-05-04

**Authors:** Manuel González-Fuente

**Affiliations:** Assistant Features Editor, Plant Physiology, American Society of Plant Biologists, USA; Faculty of Biology & Biotechnology, Ruhr-University Bochum, D-44780 Bochum, Germany

A regulated turnover of proteins is crucial for the survival of all living organisms. Autophagy is a degradation pathway responsible for the recycling of many different cellular components, from cytosolic proteins to entire organelles (Raffeiner et al. 2023). Because of its importance, autophagy is often modulated by pathogens to subvert plant defenses and favor their multiplication (Langin et al. 2020; Leong et al. 2022).

In the case of plant-virus interactions, autophagy generally acts as an antiviral defense mechanism, although in some particular cases it can also be exploited by the virus (Yang and Liu 2022). Despite their extremely limited genome size, certain viruses encode proteins able to suppress the antiviral host autophagy. So far, all of such characterized proteins come from RNA viruses (Yang and Liu 2022). DNA viruses, although less common than plant pathogens, include the large family *Geminiviridae* that currently poses an increasing threat to food security worldwide (Medina-Puche et al. 2021). DNA viral infections also trigger plant defensive autophagy (Haxim et al. 2017). Nevertheless, DNA viruses are still able to successfully infect many plant species, raising the question of how DNA viruses cope with the plant defensive autophagy.

In this issue of *Plant Physiology*, Yang et al. (2023) describe the first example of a protein from a DNA virus, the geminivirus *Cotton leaf curl Multan virus* (CLCuMuV), which suppresses the plant defensive autophagy (Figure). The authors discovered that the multifunctional viral protein C4 interacts with 2 plant proteins: the canonical autophagy component AUTOPHAGY 5 (ATG5) and EUKARYOTIC TRANSLATION INITIATION FACTOR 4A (eIF4A), a known negative regulator of autophagy (Zhang et al. 2021). In this ternary interaction, C4 enhances the association between eIF4A and ATG5, which is thought to prevent ATG5 from interacting with the rest of the canonical autophagy machinery, and so dampens autophagy.

**Figure.**
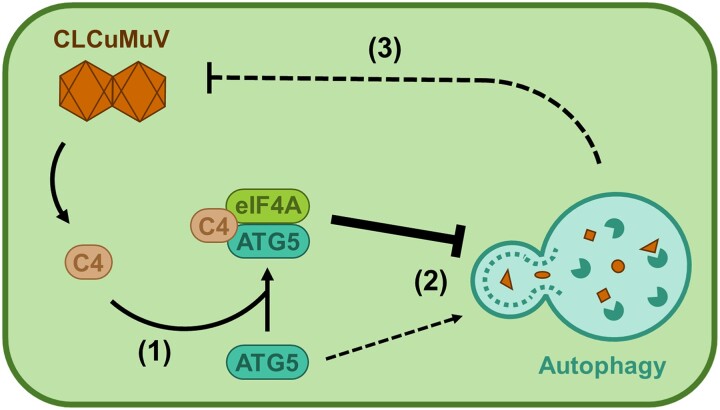
Viral protein C4 blocks the plant autophagy to promote infection. (1) CLCuMuV protein C4 interacts with eIF4A, a negative regulator of autophagy. (2) C4 enhances the association of eIF4A with ATG5, possibly preventing ATG5 from interacting with the canonical autophagy machinery, leading to suppression of plant autophagy. (3) Because plant autophagy generally acts as a defense mechanism against viruses, C4-mediated suppression of autophagy ultimately leads to an enhanced viral infection.

The authors confirmed this model by showing that a single amino acid mutant of C4 unable to interact with eIF4A does not suppress autophagy. The authors infected plants with CLCuMuV expressing either the wild-type C4 protein or the variant unable to bind eIF4A and observed that the mutant viruses replicated less and produced milder symptoms. These results indicate that the interaction of C4 with eIF4A suppresses plant defensive autophagy, facilitating viral infection.

Intriguingly, whereas the CLCuMuV protein C4 suppresses autophagy, another protein from the same virus was previously shown to induce autophagy (Haxim et al. 2017). How these 2 opposing processes impact each other and how they collectively shape the outcome of the CLCuMuV infection remains to be studied. Nevertheless, this illustrates the complexity of the molecular mechanisms underlying plant-virus and any plant-pathogen interactions, often being simultaneously and antagonistically modulated by both partners to the detriment of the other (Leong et al. 2022).

The CLCuMuV C4 protein functions span from inhibition of immune signaling to suppression of gene silencing (Medina-Puche et al. 2021). Interestingly, the authors in this work showed that the interaction of CLCuMuV C4 with the plant eIF4A, although necessary for blocking autophagy, was not required for the C4-mediated suppression of gene silencing. This highlights how, despite their extremely limited genome size, viruses maximize their chances to modulate host processes by encoding multifunctional proteins that target multiple key host components (Khorsand et al. 2020). This mirrors the properties of effector proteins from other plant pathogens such as bacteria, fungi, or nematodes, supporting convergent mechanisms to modulate host cellular processes among distinct pathogens despite their notably different lifestyles (González-Fuente et al. 2020).

This work demonstrates that, like RNA viruses, the DNA virus CLCuMuV deploys a mechanism to counteract the plant antiviral autophagy (Yang and Liu 2022). Future works will clarify how general or specific the suppression of autophagy is among other plant-pathogenic DNA viruses and whether this is mediated by similar viral proteins. Interestingly, C4 is the least conserved protein in geminiviruses, showing disparity of subcellular localizations and plant targets among different family members (Medina-Puche et al. 2021). Therefore, if other DNA viruses are indeed capable of suppressing the plant autophagy, it is possible that they would do so through alternative mechanisms.

The authors also expanded on the previously described role of eIF4A as a negative regulator of autophagy (Zhang et al. 2021). However, as its own name reflects (i.e. EUKARYOTIC TRANSLATION INITIATION FACTOR 4A), eIF4A is first and foremost a regulator of translation, facilitating the recruitment of mRNAs to ribosomes. As obligate intracellular parasites, viruses depend on the host translation machinery for the synthesis of their own proteins. Moreover, mammalian eIF4A prevents the formation of stress granules, biomolecular condensates involved in antiviral defense responses (Tauber et al. 2020). Therefore, it is likely that the interaction between CLCuMuV C4 and the plant eIF4A might have additional implications beyond suppression of autophagy.

## References

[kiad266-B1] González-Fuente M , Carrère S, Monachello D, Marsella BG, Cazalé A, Zischek C, Mitra RM, Rezé N, Cottret L, Mukhtar MS, et al Effectork, a comprehensive resource to mine for *Ralstonia*, *Xanthomonas*, and other published effector interactors in the *Arabidopsis* proteome. Mol Plant Pathol. 2020:21(10):1257–1270. 10.1111/mpp.12965 33245626 PMC7488465

[kiad266-B2] Haxim Y , Ismayil A, Jia Q, Wang Y, Zheng X, Chen T, Qian L, Liu N, Wang Y, Han S, et al Autophagy functions as an antiviral mechanism against geminiviruses in plants. Elife. 2017:6:e23897. 10.7554/eLife.23897 28244873 PMC5362266

[kiad266-B3] Khorsand B , Savadi A, Naghibzadeh M. Comprehensive host-pathogen protein-protein interaction network analysis. BMC Bioinformatics. 2020:21(1):1–22. 10.1186/s12859-020-03706-z 32912135 PMC7488060

[kiad266-B4] Langin G , Gouguet P, Üstün S. Microbial effector proteins—a journey through the proteolytic landscape. Trends Microbiol. 2020:28(7):523–535. 10.1016/j.tim.2020.02.010 32544439

[kiad266-B5] Leong JX , Raffeiner M, Spinti D, Langin G, Franz-Wachtel M, Guzman AR, Kim J, Pandey P, Minina AE, Macek B, et al A bacterial effector counteracts host autophagy by promoting degradation of an autophagy component. EMBO J. 2022:41(13):e110352. 10.15252/embj.2021110352 35620914 PMC9251887

[kiad266-B6] Medina-Puche L , Orílio AF, Zerbini FM, Lozano-Durán R. Small but mighty: functional landscape of the versatile geminivirus-encoded C4 protein. PLoS Pathog. 2021:17(10):e1009915. 10.1371/journal.ppat.1009915 34618877 PMC8496806

[kiad266-B7] Raffeiner M , Zhu S, González-Fuente M, Üstün S. Interplay between autophagy and proteasome during protein turnover. Trends Plant Sci. 2023. 10.1016/J.TPLANTS.2023.01.013 36801193

[kiad266-B8] Tauber D , Tauber G, Khong A, Van TB, Pelletier J, Correspondence RP. Modulation of RNA condensation by the DEAD-box protein eIF4A in brief RNA-RNA interactions promote formation of RNP condensates, which can be counteracted by DEAD-box proteins to curb excessive granule formation. Cell. 2020:180(3):411–426.e16. 10.1016/j.cell.2019.12.031 31928844 PMC7194247

[kiad266-B9] Yang M , Ismayil A, Gao T, Ye Z, Yue N, Wu J, Zheng X, Li Y, Wang Y, Hong Y, et al Cotton leaf curl multan virus C4 protein suppresses autophagy to facilitate viral infection. Plant Physiol. 2023. 10.1093/PLPHYS/KIAD235 37073495

[kiad266-B10] Yang M , Liu Y. Autophagy in plant viral infection. FEBS Lett. 2022:596(17):2152–2162. 10.1002/1873-3468.14349 35404481

[kiad266-B11] Zhang X , Yin Y, Su Y, Jia Z, Jiang L, Lu Y, Zheng H, Peng J, Rao S, Wu G, et al eIF4A, a target of siRNA derived from rice stripe virus, negatively regulates antiviral autophagy by interacting with ATG5 in Nicotiana benthamiana. PLoS Pathog. 2021:17(9):e1009963. 10.1371/journal.ppat.1009963 34587220 PMC8504976

